# RE-IMAGINING LTC: QUANTITATIVE AND QUALITATIVE RESULTS OF A NATIONAL MODIFIED DELPHI STUDY, IMPLICATIONS AND NEXT STEPS

**DOI:** 10.1093/geroni/igz038.2335

**Published:** 2019-11-08

**Authors:** Rosalie A Kane, Howard Degenholtz

**Affiliations:** 1 Division Health Policy & Management, School of Public Health. University of Minnesota, Minneapolis, Minnesota, United States; 2 University of Pittsburgh, Pittsburgh, Pennsylvania, United States

## Abstract

In 11/2016 Robert and Rosalie Kane began a 3-round Delphi study to re-imagine long-term care (LTC), , which took as a starting premise that LTSS in the United States fails to comport to the values and preferences of consumers. The Delphi study is “modified” from more typical Delphi designs because of 1) a sample sizes over 100, 2) an unusually broad topic--optimal LTC systems if not constrained by existing programs, financial arrangements and regulations; and 3) incorporation of new sample at each round. Round 1 asked respondents to rate and add to a list of values important to LTC< but largely was an open-ended request for respondents’ ideas, Round 2 was fielded in 6/2018 with all data collection completed by 11/2018 (the delay partly due to Robert Kane’s sudden death on March 6, 2017 and also the time needed to analyze, summarize and present the complex and detailed responses to the first round). Round 3, to be fielded in 4/2019., will provide participants with the ratings of values, principles and programmatic building blocks at Round Two, and the open-ended comment of respondents in explanation of their ratings. Each Round is analyzed cross-sectionally and can be considered a separate “virtual town square.” Ellen McCreedy and Rosalie Kane, respectively, present quantitative and qualitative results from the first two rounds. Discussants will each comment briefly from their perspectives as 1) state LTC policy developer,2) LTC university-based researcher; 3) consumer advocate, followed by audience and presenter discussion of the implications of the findings.

